# Cholera toxin: A paradigm of a multifunctional protein

**Published:** 2011-02

**Authors:** Kaushik Bharati, Nirmal K. Ganguly

**Affiliations:** *National Institute of Immunology, New Delhi, India*

**Keywords:** Adjuvant, cholera toxin, immunomodulation, immunotherapy

## Abstract

Cholera toxin (CT) was discovered exactly half a century ago by S.N. De. We have come a long way since this epoch-making discovery. Retrospectively, science had to wait a long time since Koch’s prediction of the existence of a toxin, and its actual discovery by De. CT is not just another enterotoxin that causes the signs and symptoms of the dreaded disease, cholera. It is unique in many respects, starting from its structure to its functions. CT is a multifunctional protein that is capable of influencing the immune system in many ways. It not only has remarkable adjuvant properties, but also acts as an anti-inflammatory agent, by modulating specific signal transduction pathways. Its immunomodulatory properties can be harnessed for treatment of various autoimmune disorders, and have shown great promise in the area of immunotherapeutics. CT can truly be considered as a paradigm of a multifunctional protein.

## Cholera toxin: an historical introduction

Cholera toxin (CT) is produced by *Vibrio cholerae*. As in most discoveries, a long chain of events led up to the final discovery of CT^1^. *V. cholerae* was isolated in pure culture from a stool sample originated in Egypt by Robert Koch in 1884^2^. The existence of CT was first postulated by Koch in 1886. He opined that the symptoms caused by *V. cholerae* were due to a “poison” that was secreted by the organism. However, the discovery of CT had to wait for more than half-a-century when S.N. De, in 1959, reported that cell-free *V. cholerae* culture filtrate was capable of eliciting massive accumulation of “rice-water” fluid in the ligated ileal loops of adult rabbits^3^, unequivocally demonstrating the existence of a toxin. The same year, from the Haffkine Institute in Mumbai (then Bombay), Dutta and colleagues described the production of diarrhoea in infant rabbits by a crude protein isolate from *V. cholerae* culture filtrate^4^, thereby strengthening De’s observations.

Richard Finkelstein’s group, over the following decade, subsequently isolated and purified CT^5^–^7^. Following purification, two factors were identified that possessed differing biological activities^6^* viz*., the active principle, the holotoxin (AB_5_), which was termed as “choleragen”; and the B_5_ oligomer, termed as “choleragenoid”, as it was not itself toxic, but contributed to elicit the symptoms of cholera. The heterogeneous subunit structure of CT was subsequently unequivocally demonstrated by means of biochemical techniques^8^. We now know, in intimate detail, how the CT subunits function.

The next major development was the discovery of the CT receptor by King and van Heyningen in 1973^9^. They observed that the monosialoganglioside GM1: [{Gal(β1-3)GalNac(β1-4)(NeuA-c(α2-3)Gal(β1-4)Glc}-ceramide] prevented CT from increasing the capillary permeability of rabbit skin, prevented CT from inducing accumulation of rice-water-like fluid in ligated rabbit ileal loops, and also inhibited the action of CT on the adenylate cyclase (AC) system in the guinea pig small intestine.

Following these landmark discoveries, many unique functions of CT were discovered that would pave the way towards finding suitable medical applications. This review will address the issue of the multi-functionality of this remarkable protein.

## Cholera toxin: from structure to function

CT is made up of two types of subunits. The larger A subunit (240 amino acids; MW 28 kD) is located centrally, while the five B subunits (103 amino acids; MW 11 kD each; aggregate MW ~56 kD) are located peripherally. The A subunit of CT has over 82 per cent sequence identity with the *Escherichia coli* heat-labile enterotoxin (LT), while the B subunits of the former shares over 83 per cent sequence identity with the latter^10^. The elucidation of the three-dimensional structure of CT was a major achievement to enhance our understanding of this remarkable toxin^11^,^12^. The three-dimensional structure of CT further corroborated the sequence data that it shared a similar structure to LT, the crystal structure of which had been determined a few years earlier^13^,^14^. The A subunit consists of two domains (A1 and A2). In both the toxins, the upper A1 domain of the wedge-shaped A subunit is held above the plane of the doughnut-shaped pentameric B subunits by the tethering A2 domain, which in case of CT, is an alpha helix for almost its entire length. The carboxy-terminal of the A2 passes through the opening created by the doughnut arrangement of the B subunits. The four carboxy-terminal residues of the A2 chain are Lys-Asp-Glu-Leu (K-D-E-L).

**Fig. 1:**
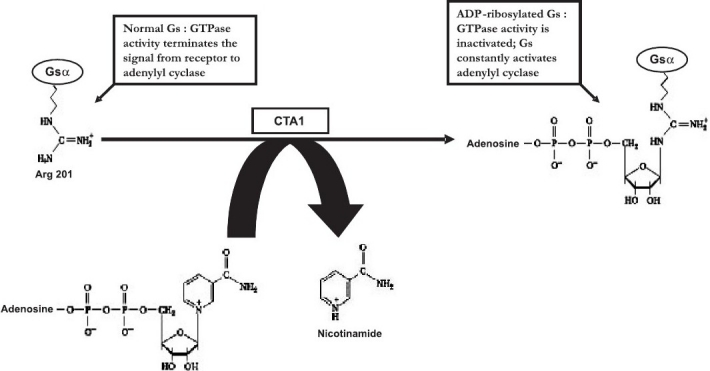
ADP-ribosylation. The 22kD A1 domain of CT (CTA1) catalyzes the transfer of the ADP-ribose moiety of NAD^+^ to an Arginine residue (Arg^201^) of the α subunit of Gs, leading to defective regulation of adenylyl cyclase and overproduction cAMP.

Cholera toxin, by acting as a classical A-B type toxin, leads to ADP-ribosylation of G protein, and constitutive activation of AC, thereby giving rise to increased levels of cyclic AMP within the host cell (Fig. 1). As a result, electrolyte imbalance occurs due to a rapid efflux of chloride ions by the cystic fibrosis trans-membrane conductance regulator (CFTR), decreased influx of sodium ions, leading to massive water efflux through the intestinal cells, thereby causing severe diarrhoea and vomiting, the cardinal clinical signs of cholera. Diarrhoea, if untreated, leads to severe dehydration, electrolyte abnormalities and metabolic acidosis^15^, almost inevitably resulting in death.

The toxic action of CT is initiated by binding of its B subunits to the high-affinity monosialoganglioside GM1 receptors. Each B subunit monomer has a binding site for GM1. Moreover, a single amino acid from a neighbouring B subunit also plays an important role in binding^16^, explaining the much higher binding affinity of the CTB pentamer, as compared to that of the CTB monomer. Endocytosis of CT may follow one of three pathways: (*i*) lipid raft/caveolae mediated endocytic pathway, (*ii*) clathrin mediated endocytic pathway, or (*iii*) ADP-ribosylation factor 6 (Arf6)-associated endocytic pathway. CT then travels to the endoplasmic reticulum (ER) in a retrograde fashion. After reaching the ER, the A subunit of CT (CTA) dissociates from its B subunit (CTB). Earlier studies indicated that a functional Golgi apparatus was essential^17^ in this transportation pathway, but later studies showed that the Golgi system was not mandatory^18^. It was initially thought that the KDEL signal, present at the carboxy-terminal of CTA2, which is a classical eukaryotic signal for ER retention, was essential for retrograde trafficking. However, mutagenesis and blocking studies have indicated that this signal is not essential for retrograde transport, but rather, serves for the retrieval of the dissociated CTA from the Golgi apparatus to the ER^17^,^18^. This observation has been strengthened by the fact that CTB, which does not possess a KDEL signal, is also transported to the ER in a retrograde fashion^19^. The ADP ribosylation activity of CT resides in CTA1. Hence, entry of CTA1 into the cytosol is a crucial step in the intoxication process. This involves the ER-associated degradation pathway, or degradasome, the function of which is to retrieve misfolded proteins from the ER for their degradation in the cytosol. CTA1 escapes degradation in spite of passing through the degradasome, presumably because of its low lysine content, an essential marker for ubiquitination^20^. During transport through the degradasome CTA1 unfolds and refolds, which involves reduction by protein disulphide isomerase and reoxidation by Ero1^21^. CTA1, upon entry into the cytosol, catalyzes the ADP-ribosylation of the trimeric Gsα component of AC (Fig. 1). This leads AC to remain in its GTP-bound state, resulting in enhanced AC activity and increased intracellular cAMP concentrations. High levels of cAMP start a cascade that eventually lead to the severe clinical manifestations of cholera, as has been highlighted above.

## WO7:A novel cholera toxin

During an outbreak of cholera in Warangal, Andhra Pradesh, *V. cholerae* WO7 (serogroup O1) strain was isolated from patients with diarrhoea. The interesting point about this strain was the fact that it produced an extracellular toxin (WO7) despite the absence of *ctx, zot*, and *ace* genes from its genome. The purified WO7 toxin caused fluid accumulation in ligated rabbit ileal loops, elongation of Chinese hamster ovary cells, rounding of Vero cells and agglutination of freshly isolated rabbit erythrocytes. The purified toxin was highly specific for antisera raised in rabbits against the purified toxin, as revealed by immunodiffusion and immunoblot assays. However, the toxin did not cross-react with either CT or LT, in these systems. The WO7 toxin agglutinated GM1-coated erythrocytes, indicating that GM1 could be its receptor. The enterotoxic activity of the WO7 toxin was found to be 10-fold greater than that of CT, but could be neutralized by antisera raised in rabbits against the purified toxin^22^. A model was subsequently proposed to explain the mode of action of the WO7 toxin at the intracellular level, implicating Ca^2+^, cAMP, inositol triphosphate and protein kinase C in playing critical roles^23^. It was also found that upon WO7-toxin stimulation of enterocytes, an increase in the level of reactive oxygen species was noted, that correlated with a simultaneous decrease in the levels of antioxidants such as catalase and superoxide dismutase. The levels of reactive nitrogen intermediates were also found to be high in WO7-toxin treated enterocytes, indicating that the toxin played a role in altering mucosal permeability. It was concluded that significant increase in the levels of second messengers, coupled with a decrease in antioxidant defenses were key factors in mediating the enterotoxic action of the toxin. The ability of serogroup O1 strain *V. cholerae* WO7 to produce a toxin 10 times more potent than CT, in spite of the absence of *ctx, zot* and *ace* genes, is a cause for concern and the foregoing findings warrant further in-depth studies of this strain.

## Adjuvant action of cholera toxin

That CT possessed adjuvant activity, was first reported in the early seventies^24^. Injection of sheep red blood cells (SRBCs) with CT into mice resulted in a 13-fold increase in the levels of systemic anti-SRBC antibody titres. Moreover, transcutaneous immunization was made possible by simply mixing CT with various antigens, such as tetanus toxoid, diphtheria toxoid or bovine serum albumin (BSA) and applying on the skin of mice^25^. The adjuvant activity of CT may be attributed to the enhanced antigen presentation by various types of antigen presenting cells (APCs), such as macrophages, dendritic cells (DCs) and B cells. In B cells, both recombinant CT (rCT) and recombinant CTB (rCTB) promote isotype differentiation that leads to increased IgA formation. It has been suggested that both enzymatic activity and receptor binding contribute to the stimulatory effects of the toxin^26^. CT has also been reported to upregulate various cell surface molecules such as co-stimulatory molecules and chemokine receptors in murine and human DCs as well as in other APCs^27^,^28^. CT also stimulates the secretion of IL-1 from macrophages, which enhances their APC function^29^. IL-1 itself is also an efficient mucosal adjuvant. The polarity of the immune response generated by CT is a matter of debate. While some studies indicate that CT-treated DCs prime naïve T cells *in vitro* and drive them towards a Th2 phenotype^30^, others have reported the toxin to induce a mixed Th1/Th2 type of immune response^28^. In contrast to its stimulatory effect on B cells and APCs, rCTB induces apoptosis of CD8+ T cells^31^. Importantly, depletion of CD8+ T cells by rCTB has been observed *in vivo*^32^.

CT strongly potentiates the immunogenicity of most antigens, whether these are linked to or simply admixed with the toxin, provided that the antigen is given at the same time and at the same mucosal surface as the toxin. There are various antigens towards which CT exhibits adjuvant activity (Table I)^33^–^47^.

**Table I:** Antigens towards which CT or its subunits have adjuvant activity

Antigen	Route	Reference
Keyhole Limpet	peroral (p.o.)	33,34
Haemocyanin (KLH)	intranasal (i.n.) intravenous (i.v.)	35
Ovalbumin (OVA)	i.n. i.v.	35
Antigen MI (AgMI) from *Streptococcus mutans*	p.o.	36
Influenza virus	p.o.	37
*Pseudomonas aeruginosa polysaccharide*	p.o.	38
Sendai virus	p.o. i.n. subcutaneous (s.c.)	39
Tetanus toxoid (TT)	p.o.	40
Killed *Campylobacter*	p.o.	41
*Toxoplasma gondii* sonicate (Tso)	p.o.	42
Respiratory syncytial virus glycoprotein F (RSV gF)	i.n.	43
*Helicobacter pylori* recombinant Urease (*H. pylori* rUrease)	p.o.	44
Soluble worm antigen preparation (SWAP) from *Schistosoma mansoni*	intradermal (i.d.)	45
*T. gondii* surface antigen 1 (SAG1)	i.n.	46
Pneumococcal surface protein A (PspA) from *Streptococcus pneumoniae*	p.o.	47

### 

#### Development of non-toxic derivatives of CT as mucosal adjuvants

Although animal studies were highly impressive, it soon became evident that the CT holotoxin was much too toxic for use in humans. Hence, to circumvent the toxicity problems associated with the holotoxin, efforts were directed towards developing non-toxic recombinant derivatives of CT. CTB was an obvious choice as an adjuvant, as it was non-toxic. However, CTB proved to be a much poorer adjuvant than its holotoxin counterpart. Coupling the antigens to CTB increased the immunogenicity appreciably as it facilitated receptor-mediated uptake and subsequent presentation by the APCs. Some examples of antigens expressed as CTB fusions are given in Table II^48^–^50^.

**Table II:** Some foreign antigens expressed as CTB fusions

Antigen	Immunogenicity	Reference
HIV-1 envelope protein gp120	Serum anti-gp120 IgG (in mice)	48
A decapeptide highly homologous to the heat-stable enterotoxin of *E. coli* (STa)	Serum anti-STa IgG (in rabbits)	49
Serine-rich *Entamoeba histolytica* protein (SREHP)	Serum anti-SREHP IgG, IgA; stool and bile IgA (in mice)	50

Site-directed mutagenesis was one approach to generate CT mutants with reduced toxicity but retaining significant adjuvanticity. Initial studies indicated that if the ADP-ribosylating activity (the toxic property of CT) was absent in the mutant derivatives, the adjuvant function was also lost^51^. However, subsequent studies indicated that the ADP-ribosyltransferase activity was not essential for the adjuvant function, and that separation of this toxic component in many instances did not hamper the adjuvanticity appreciably^52^. Some examples of non-ADP-ribosylating mutants exhibiting immunogenic and adjuvant activity are given in Table III^53^–^55^. Partial blocking of the ADP-ribosylation site can be achieved by adding peptides to the amino terminal of CTA1 that produces steric hindrance on the enzymatically active site, thereby reducing the toxicity^56^. In yet another strategy, CTA1 was linked to a cell-binding domain, having affinity for APCs, derived from *Staphylococcus aureus* protein A (CTA1-DD). This hybrid specifically targets the B cells and has been found to work well when administered intranasally, but not so well when administered orally. This limitation has been overcome by fusing CTA1-DD to immune- stimulating complexes. Oral immunization with the CTA1-DD-ISCOM fusion complex generated both systemic and mucosal responses with Th1/Th2 bi-polarity^57^. Synthetic oligodeoxynucleotides (ODNs) containing unmethylated ‘CpG’ motifs have been successfully employed as systemic adjuvants and represent a promising mucosal adjuvant. Linking CpG ODNs to CTB (CpG ODN-CTB conjugate) represents a novel strategy that is capable of activating APCs *in vitro* and stimulating both T cell and antibody responses *in vivo*^58^.

**Table III:** Immunogenicity and adjuvant activity of some non-ADP-ribosylating CT mutants

Antigen	Mutation	Animal (route)	Enzymatic and biological activity	Immunogenicity (antibody type)	Adjuvant activity	Reference
OVA	E112K S61F	Mice (s.c.)	–	+ (IgG; IgE)	+	53
OVA, TT, Influenza virus	S61F	Mice (i.n.)	–	+ (IgG; IgA)	+	54
OVA, Fragment C of TT	S63K	Mice (i.n.)	–	± (IgG; IgA) (negligible)	± (negligible)	55
	P106S		±	± (IgG; IgA)	±	

E112K=Glu to Lys at position 112; S61F=Ser to Phe at position 61; S63K=Ser to Lys at position 63; P106S=Pro to Ser at position 106

## Cholera toxin and immunomodulation

In addition to its adjuvant properties, CT has been recognized as a potent immunomodulator. The immunomodulatory function of CT can be attributed to three basic properties of the toxin itself: (*i*) ability to act as a true enterotoxin by exhibiting stability in the harsh enteric environment, (*ii*) ability to bind, via the B subunits, to specific GM1 ganglioside receptors present in the gut mucosa as well as APCs, thereby facilitating antigen uptake and presentation, and (*iii*) inherent immunomodulatory properties of the toxin itself.

**Fig. 2:**
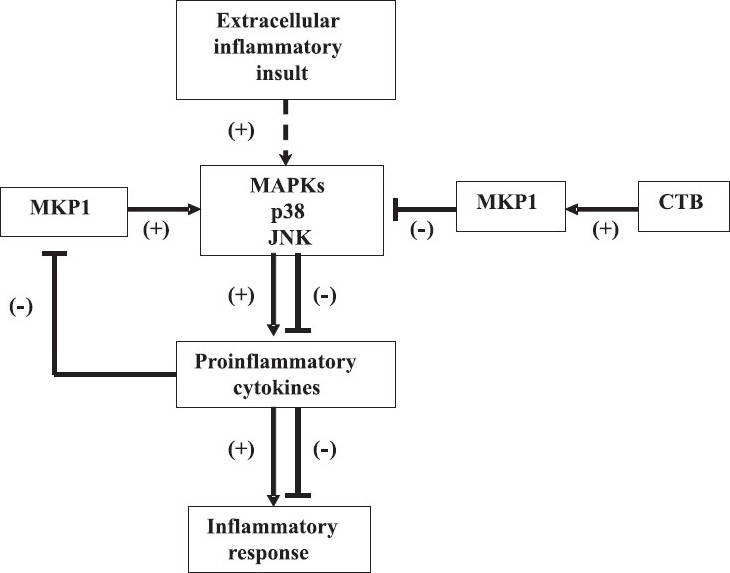
Molecular basis of immunomodulation by CTB. MKP1 expression plays a central role in immunomodulation by immunomodulatory agents like CTB. Extracellular inflammatory insult activates MAPK, p38 and JNK pathways, leading to generation of proinflammatory cytokines, resulting in an inflammatory response. Proinflammatory cytokines have an inhibitory effect on MKP1, which leads to further activation of the inflammatory response pathway (left side of diagram). CTB, as well as other anti-inflammatory agents augment MKP1 expression, which leads to inhibition of the pro-inflammatory cascade, thereby attenuating the inflammatory response (right side of diagram). MAPK: mitogen-activated protein kinase; MKP1: MAPK phosphatase-1; JNK: JUN N-terminal kinase.

*V. cholerae*activates the innate immune response through its lipopolysaccharide (LPS) binding to Toll-like receptor 4 present on the surface of APCs such as macrophages, leading to the release of proinflammatory cytokines like TNFα by activation of a signal transduction cascade. However, prior treatment of macrophages with CTX suppresses TNFα production in response to LPS, by up to 80 per cent. Since nitric oxide (NO) synthesis requires signaling by TNFα, CT treatment simultaneously reduces the production of NO^59^. Importantly, it has been found that similar effects can be produced by the CTB alone^60^, indicating that the immunomodulatory activity might reside in CTB and do not require the catalytically active A subunit for this function. The underlying mechanism by which CTB exerts its immunomodulatory action has been dissected out by studies involving the mitogen activated protein kinase (MAPK) signal transduction pathway. It has been found that CTB induces MAPK phosphatase-1 (MKP1) expression and significantly inhibits Janus kinase and p38 activation, thereby leading to a substantial attenuation of TNFα and IL-6 production by LPS-stimulated macrophages^61^. It has been suggested that induction of MPK1 expression could be a potential underlying mechanism for the immunomodulatory action of CTB (Fig. 2).

## Cholera toxin B subunit for mucosal tolerization and immunotherapy

CT is capable of inducing mucosal immunological tolerance. Initial experiments revealed that when mice were fed with CTB-conjugated SRBC, there was an abrogation in the delayed-type hypersensitivity and IgE antibody responses upon systemic immunization with SRBCs^62^. Subsequent studies revealed that the ability of CTB to suppress inflammatory responses in an antigen-specific manner could hold potential for treatment of autoimmune diseases. Animal models of various autoimmune diseases were used in these studies. Thus, CTB conjugated to myelin basic protein was shown to prevent the development of experimental allergic encephalomyelitis, the rat equivalent of multiple sclerosis that affects humans^63^. Moreover, a CTB-insulin conjugate delayed the onset of insulin dependent diabetes mellitus in nitric oxide deficient mice^64^. More importantly, unconjugated rCTB has also been found to induce long-lasting protection from diabetes in NOD mice^65^. Investigation of the mechanism of oral tolerization by CTB has revealed that, besides increasing the uptake of antigen across the mucosal barrier and presentation by APCs, regulatory T cells play a prominent role in the process^66^.

One of the major success stories of oral tolerance induction with CTB-antigen conjugates relates to the work on Behcet’s disease (BD). BD is a multi-system inflammatory disorder with uveitis being a major complication. Following the identification of a BD-specific peptide (p336-351) within the human 60kD heat-shock protein, it was found that oral tolerization could be induced in Lewis rats by linking the peptide to rCTB, effectively reducing the incidence of uveitis from 65.8 to 16.7 per cent^67^. This study was followed by a phase I/II clinical trial in a selected number of patients. Oral tolerization with the BD-peptide-rCTB conjugate was attempted in patients with BD for preventing relapses of uveitis. The conjugate was administered thrice-weekly for 12 to 16 wk, which allowed withdrawal of all immunosuppressive drugs in 5 of the 8 patients or 5 of 6 patients (who were free of disease activity prior to tolerization) without a relapse of uveitis. The clinical manifestations were correlated with immunological findings^68^. The positive findings of the preceding study have possibly fuelled a recent clinical trial in 15 patients suffering from Crohn’s disease to address the issues of safety and short-term efficacy of rCTB in this particular autoimmune condition. Treatment with rCTB was found to be safe and well tolerated, with approximately 40 per cent of patients responding to treatment^69^.

## Cholera toxin B subunit for cholera vaccine development

Enteric infections accompanied by diarrhoea are a leading public health problem, particularly in the developing countries. Enterotoxin-producing bacteria are a leading cause of diarrhoea, of which *V. cholerae* accounts for the most severe disease, often in the form of epidemics. Most probably, the greatest contribution towards alleviating this age-old disease is the incorporation of rCTB itself in the vaccine preparation. Of the internationally licensed oral cholera vaccines, the most widely used vaccine, DUKORAL^™^ (SBL Vaccines, Sweden), consists of a mixture rCTB and heat or formalin-inactivated *V. cholerae* O1 whole cells of different serotypes and biotypes. This vaccine has been shown to be safe and effective in large-scale clinical trials.

## Conclusion

CT is a multifunctional protein that is truly remarkable in many respects. CT was initially thought to be just another enterotoxic protein that caused the life-threatening symptoms of cholera but further research revealed that this toxin possessed many unique features. Besides its unique structure, mode of intracellular trafficking, and ADP-ribosyltransferase activity, the toxin possesses a multifaceted character with regard to functionality. The major contribution of this unique toxin has been in the field of Immunology functioning as an effective adjuvant or an immunomodulator and holding promise in the area of therapeutics against various types of autoimmune diseases. Moreover, this toxin has a remarkable property for downregulating inflammatory reactions and the molecular mechanisms of its action could open avenues towards development and design of new anti-inflammatory agents for modulating various immune disorders in the foreseeable future.
